# COCO enhances the efficiency of photoreceptor precursor differentiation in early human embryonic stem cell-derived retinal organoids

**DOI:** 10.1186/s13287-020-01883-5

**Published:** 2020-08-24

**Authors:** Deng Pan, Xi-Xi Xia, Heng Zhou, Si-Qian Jin, Yang-Yan Lu, Hui Liu, Mei-Ling Gao, Zi-Bing Jin

**Affiliations:** 1 Laboratory of Stem Cell & Retinal Regeneration, Institute of Stem Cell Research, Division of Ophthalmic Genetics, The Eye Hospital, Wenzhou Medical University, Wenzhou, 325027 China; 2 Beijing Institute of Ophthalmology, Beijing Tongren Eye Center, Beijing Tongren Hospital, Capital Medical University, Beijing Ophthalmology & Visual Science Key Laboratory, Beijing, 100730 China; 3 Beijing Advanced Innovation Center for Big Data-Based Precision Medicine, Beihang University & Capital Medical University, Beijing Tongren Hospital, Beijing, 100730 China; 4 School of Basic Medical Sciences, Wenzhou Medical University, Wenzhou, 325035 China

**Keywords:** Retinal organoid, 3D, COCO, CRX, Photoreceptor precursor, Fluorescent labeling, Cone

## Abstract

**Background:**

Significant progress has been made in cell replacement therapy for neural retinal diseases using retinal cells differentiated from human pluripotent stem cells. Low tumorigenicity and the ability to mature to form synaptic junctions make precursor cells a promising donor source. Here, we attempted to improve the yield of photoreceptor precursor cells in three-dimensional retinal organoids from human embryonic stem cells (hESCs).

**Methods:**

A CRX-tdTomato-tagged hESC line was generated to track retinal precursors in 3D retinal organoids. COCO, a multifunctional antagonist of the Wnt, TGF-β, and BMP pathways, was employed to 3D organoid differentiation schemes for enhanced photoreceptor precursor cells. Organoid fluorescence intensity measurement was used to monitor retinalization tendency with the number of precursors further checked by flow cytometry. Signature gene expression during organoid differentiation were assessed by qPCR and immunocytochemistry after COCO supplementation.

**Results:**

CRX-positive cells can be spatiotemporally tracked by tdTomato without affecting retinalization during retinal organoid differentiation. Fluorescence intensity of organoids, which turned out highly consistent with flow cytometry measurement, allowed us to determine the differentiation efficiency of precursors during organoid culturing directly. Using COCO as an auxiliary supplement, rather than alone, can yield an increased number of photoreceptor precursors in the early stage of organoid differentiation. Over a longer time-frame, photoreceptor precursors enhanced their fate of cones and decreased fate of rods after treatment with COCO.

**Conclusions:**

Tracing with the CRX-reporter system showed that in retinal organoids derived from human pluripotent stem cells, COCO increased the differentiation efficiency of photoreceptor precursors and cones.

## Introduction

The majority of retinal cell types can be formed by in vitro differentiation of pluripotent stem cells (PSCs). Employing healthy cells differentiated from PSCs to replace damaged cells in diseased eyes through cell transplantation has revealed favorable prospects in laboratory and clinical studies [1–3]. Among alternative cell types used for retinal regeneration, cone photoreceptors and precursors have received widespread attention for the purpose of photoreceptor replacement [4–8]. Therefore, improving the differentiation efficiency of photoreceptor precursor cells during retinalization to supply an unlimited source for cell transplantation will undoubtedly provide valuable applications in the future.

To enable integration with host retinal cells, it is necessary to provide cells that are similar to those in the physiological situation [8, 9]. Such cells can present efficient and robust integration, which is a prerequisite for effective cell therapy. Compared with the single cell type obtained from most 2D differentiation, the multiple types of retinal cells obtained in a 3D differentiation system form well-laminated and properly stratified structures similar to the real retina, which are obviously closer to the physiological state [10, 11]. However, increasing the ratio of a certain type of cell is challenging in 3D culturing because of existing inherent cellular programs that form retinal organoids with self-organization. Nonetheless, several successful cases have been reported by artificially transforming the cell tendency in the natural state of organoids through external signal pathway intervention. For example, inhibiting Notch signaling during the early period of differentiation accelerates the transformation of progenitor cells into precursor cells, while eliminating the inhibition of GSK3 FGFR promotes the transition of RPE cells to the neural retina [12, 13]. However, it is unknown whether an earlier stage of intervention during stem cell differentiation would achieve more effective production of photoreceptor precursor cells.

Fluorescent labeling of target cells during in vitro differentiation has played a huge role in organoid research [14]. With the help of fluorescently labeled genes of interest, it is possible to determine the optimal culture conditions to promote the formation of organoids, to perform real-time imaging of 3D organoids and to track abnormal mitotic characteristics of targeted cells in pathogenesis [15–18]. *CRX* is a basic homeobox gene with predominant expression in postmitotic photoreceptor precursors, regulating both cone and rod fate and maturation [19]. In this study, we used *CRX* as a target gene to construct a fluorescence-labeled embryonic stem cell (ESC) line to trace photoreceptor precursor cells during 3D differentiation. With this system, CRX-positive cells can be spatiotemporally tracked without affecting retinalization during 3D differentiation. We then employed COCO, a reported multifunctional antagonist of the Wnt, TGF-β, and BMP pathways, to our 3D retinal organoid differentiation. Our results demonstrate that COCO can work with Wnt inhibitors in the original differentiation system to increase the number of photoreceptor precursors in the early stage of differentiation.

## Methods

### Generation of the knock-in hESC line

**Fig. 1:**
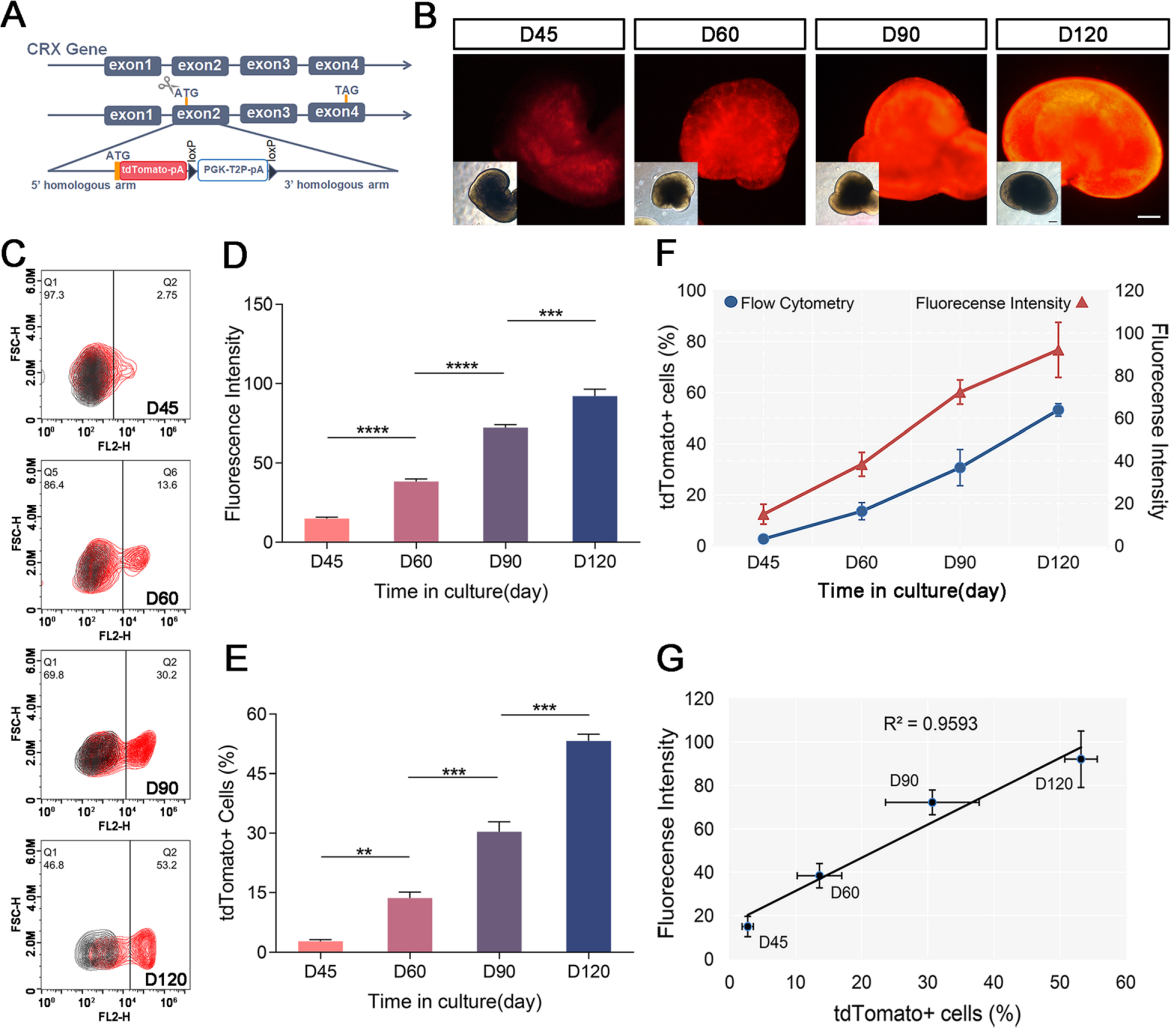
Generation of reporter knock-in CRXp-tdTomato cell line. **a** Schematic diagram showing the targeting strategy of the insertion site. tdTomato cDNA sequence was fused in-frame into CRX behind start codon. **b** Overall 3D organoids fluorescent and bright field images on D45, D60, D90, and D120. A typical fluorescence intensity increasement along with differentiation time is presented. **c** Representative flow cytometry analysis in D45, D60, D90, and D120 organoids. Black and red represent the organoids derived from control and knock cell line, respectively. **d** Overall fluorescence intensity of organoids quantified by ImageJ, data are presented as the mean ± SEM (*n* = 27, 14, 11, and 10, respectively). **e** Proportion of tdTomato-positive cells in 3D organoids counted by flow cytometry, data are presented as the mean ± SEM (*n* = 6, 6, 8, and 3, respectively). **f** Comparison of increasement tendency between flow cytometry analysis and fluorescence intensity. **g** Relationship between fluorescence intensity and the number of tdTomato-positive cell in 3D organoids

Our gene-targeting strategy is illustrated in Fig. 1a. To introduce the exogenous gene into the H9 cell line, a vector plasmid was designed and constructed. The plasmid contained the cDNA of tdTomato, and a PGK promoter-driven puromycin-resistance selection cassette flanked by loxP sites was inserted downstream of tdTomato. The sgRNA sequences targeting CRX gene exon 2 at the start codon ATG were designed and introduced into the plasmid described above. The constructed vectors were delivered into hESCs (H9 line, Biocytogen, Beijing) by electroporation and then were selected with puromycin. Finally, the positive clones were identified by PCR and sequencing.

### Culture of hESCs

Human ESCs were maintained on Matrigel-coated (Cat. #356231; BD Corning, United States) dishes in mTeSR-E8 medium (Cat. #05940; Stemcell Technologies, Canada). The cells were passaged with 0.5 μM EDTA for dissociating colonies, resuspended with mTeSR-E8 medium containing 10 μM Y27632 to reduce cell death when clones reached approximately 80% confluence.

### Differentiation of 3D retinal organoids

3D retinal organoids were generated as described previously [20]. Human ESCs were disrupted to form a suspension of single cells that were allowed to quickly aggregate in low-cell adhesion 96-well plates with V-bottomed conical wells (9000 cells per well in 100 μl). Cells were maintained in 20% O_2_ and 5% CO_2_ atmosphere. In COCO-supplemented experiments, COCO at a final concentration of 30 μM was added to the culture medium from day 0 to day 12 or day 30.

### FACS analysis

Organoids derived from CRXp-tdTomato and H9 were washed with DPBS and dissociated by trypsin EDTA (Life Technology) and DNase I (Sigma) for 8 min at 37 °C to generate single cells. After inactivation with a cocktail containing DPBS, FBS (Cat. #04-002-1A; Biological Industry), and DNase I, the single cells were suspended in the mixed solution for filtering. The resulting cells were analyzed by FACS Aria (BD Biosciences). The results were subsequently analyzed using FlowJo software.

### Real-time quantitative PCR

For qPCR, total RNA from 3 to 5 organoids from three independent differentiation experiments was extracted using TRIzol Reagent (Cat. #15596018; Invitrogen) in accordance with the manufacturer’s instructions. Total RNA was reverse-transcribed into cDNA using M-MLV Reverse Transcriptase (Promega; Cat. #M1705) following the experimental protocol. Quantitative PCR was performed on the cDNA samples using a real-time PCR system (LightCycler 96 System; Roche, Mannheim, Germany) with a master mix (FastStart Universal SYBR Green Master [ROX]; Roche). Expression levels of genes of interest were calculated assuming a PCR efficiency of 100%, and they were normalized to the housekeeping genes beta-actin or GAPDH.

### Fluorescence intensity quantification of retinal organoids

During retinal organoid differentiation, fluorescence images of organoids were captured with an inverted fluorescence microscope (Nikon TE2000; Japan) using the same filters and magnification. In addition, the organoids that were imaged were selected randomly. The mean fluorescence intensity of each organoid was quantified by ImageJ software with the default parameter settings. Briefly, imported image was firstly converted to 8-bit. Then, the “threshold” was adjusted to delineate measured area for gray value analysis. The “mean” value in the results panel was used to be the mean fluorescence intensity of the measured area.

### Pearson’s correlation coefficient analysis

Immunostaining of retinal organoid cryosections was performed as described elsewhere. Immunofluorescent images were taken randomly for each timepoint with a × 40 water objective, and the colocalization correlation between CRX-positive signals and tdTomato fluorescent signals was analyzed by the “Coloc2” plugin of ImageJ software. Briefly, after imported into ImageJ software, the multi-color fluorescence image was split channels by the “Color-Split Channels” tool. The two channels were converted to 8-bit to be analyzed. Then, “Coloc 2” plugin was used to colocalization analysis between two channels followed by online tutorial (https://imagej.net/Coloc_2). “Pearson’s correlation coefficient” in the result panels was used for further statistical analysis.

### Statistical analysis

Statistical analysis was performed with GraphPad Prism version 5.0 and Microsoft Excel. The standard errors of means (SEM) or the standard deviation (SD) were calculated. Statistical significance was tested using an unpaired two-tailed *t*-test. Statistically significant differences were defined as *P* < 0.05.

## Results

### Correlation between fluorescence intensity and number of fluorescent cells in retinal organoids

To intuitively and quickly judge whether the differentiation scheme effectively improved the production of photoreceptor precursor cells, we first constructed a cell line based on ESC H9 with CRX labeled by tdTomato. PCR was used to verify sequences upstream and downstream of the insertion site, as well as unmodified alleles (Figure S1). Karyotype analysis revealed no chromosomal aberrations in the CRXp-tdTomato line. The expression of a-fetoprotein (AFP), a-sarcomeric actin (α-SMA), and glial fibrillary acidic protein (GFAP) showed endoderm, mesoderm, and ectoderm differentiation potential, respectively. Together with the coloring of alkaline phosphatase staining, the CRXp-tdTomato line exhibited a defining property of pluripotency.

We used a classic 3D differentiation method by interfering with the Hedgehog and Wnt signaling pathways to guide ESCs toward retinal differentiation. Organoids began to show fluorescent signals around D28 of differentiation. We tracked the fluorescence expression during organoid differentiation with a wide-field fluorescence microscope. At D45, D60, D90, and D120 of differentiation, we acquired organoid images under the same parameters and then quantified fluorescence intensity by ImageJ. The results revealed a gradual increase in fluorescence intensity passed by differentiation (Fig. 1b, d).

We further performed flow cytometry on organoids at the same day of differentiation to determine the exact proportion of fluorescent cells. Figure 1 c shows that the ratio of fluorescent cells at D45, D60, D90, and D120 was 2.75%, 13.6%, 30.2%, and 53.2%, respectively. We further analyzed the relationship between the fluorescence intensity of organoids and the proportion of fluorescent cells. The results showed a high correlation between the two measurements (Fig. 1f, g). Compared with the flow cytometry analysis, the overall fluorescence intensity decreased slightly at D120 (exhibited as a reduced slope). The limited intensity increase was attributed to overexposure of fluorescence intensity on the D120 organoids with the same imaging parameters. Obviously, the overall fluorescence intensity accumulation was caused by the increase in the number of fluorescent cells. We concluded that this reporter strategy could help us directly judge the production of photoreceptor precursor cells.

### Effective fluorescent labeling of photoreceptor precursor cells during 3D differentiation

**Fig. 2:**
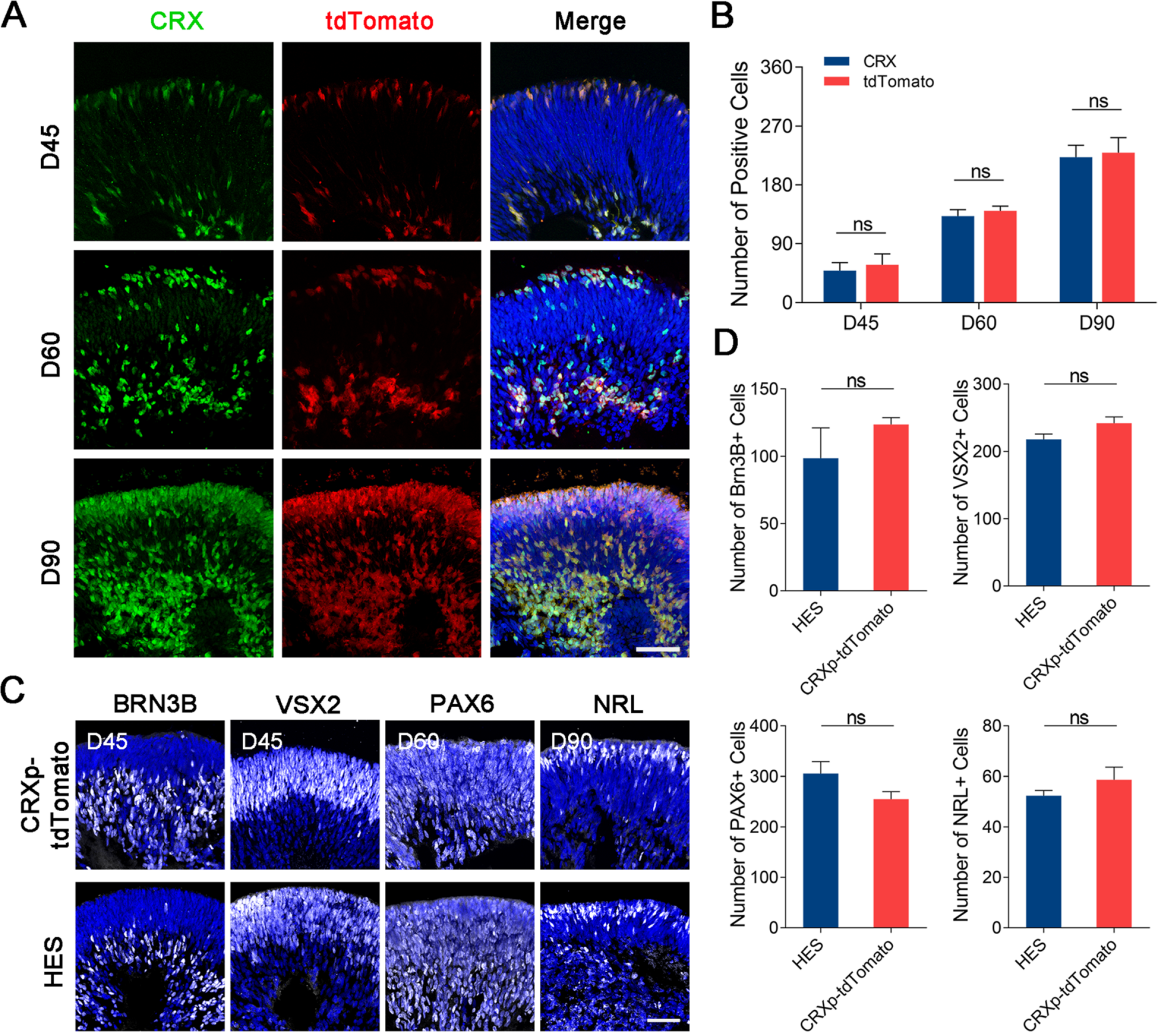
tdTomato accurately traced photoreceptor precursor cells in 3D retinal organoids. **a** Immunocytochemistry with anti-CRX showing high correlation between tdTomato and CRX in D45, D60, and D90 organoids. Scale bar, 50 μm. **b** Quantification of anti-CRX-positive cells and tdTomato-positive cells at different stages of differentiation. **c** Immunocytochemical analysis between H9 and CRXp-tdTomato after retinal organoid differentiation indicates an undisturbed progenitor, ganglion cell, and rod development. Scale bar, 50 μm. **d** Quantification of ganglion, progenitor, and rod cells in D45, D60, and D90 organoids

To confirm the relationship between fluorescence and CRX expression, we visualized endogenous CRX using immunohistochemistry. The results showed that in organoids on D45, D60, and D90, anti-CRX cells were not significantly different from tdTomato-positive cells (Fig. 2a, b). In addition, the pixel intensity from two image channels correlated well with each other from D45, D60, and D90 organoids (Pearson’s correlation coefficient = 0.79 ± 0.02, 0.86 ± 0.06, and 0.86 ± 0.04, respectively). These results indicated that tdTomato spatiotemporally followed the expression of *CRX*. Considering the characteristic localization of CRX in the retina, we believe that tdTomato successfully traced photoreceptor precursor cells and mature photoreceptor cells in these 3D retinal organoids.

CRXp-tdTomato cells showed the same organoid yield as H9 cells, suggesting that genetic modification of H9 did not affect its retinalization process (data not shown). To further investigate whether fluorescent knock-in affected the expression of other genes in retinal organoids, we performed immunostaining for several signature gene products at different stages of organoids (Fig. 2c). VSX2-positive cells on D45 and PAX6-positive cells on D60 organoids were expressed at equivalent levels between the H9 and CRXp-tdTomato groups, suggesting unaffected retinal progenitor cell development after reporter knock-in (Fig. 2d). In both cell lines, BRN3B-positive cells (representing ganglion cells) were distributed on the inner side of organoids and were expressed at the same high level in the early stage of differentiation (45 days), which was consistent with the natural characteristics of ganglion cells. Moreover, there was no difference in the spatial distribution and NRL-positive cell numbers on D90 organoids, representing a normal initial state of rod cells at the middle stage in the two groups. Therefore, tdTomato knock-in did not impact the production of early progenitor cells, ganglion cells or rods along with 3D retinal recapitulation.

### COCO temporally enhances the differentiation efficiency of photoreceptor precursor cells

**Fig. 3:**
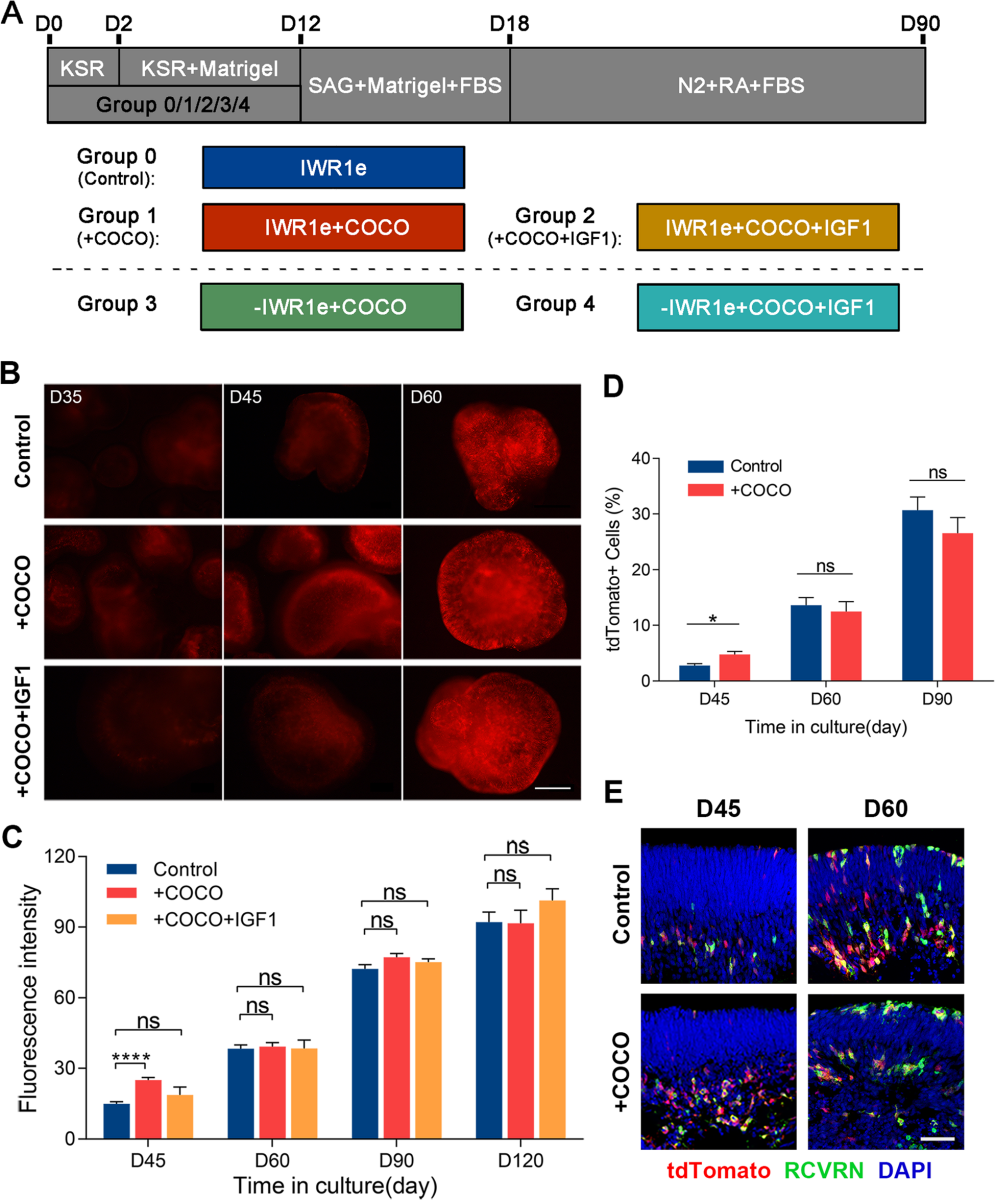
Acceleration of photoreceptor precursor cells after COCO supplement. **a** Schematic illustration outlining the differentiation protocol to improve precursor cells with COCO. **b** Fluorescent organoids inducing efficiency supplied by COCO and COCO with IGF1 at different developmental stages. Scale bar, 100 μm. **c** Fluorescence intensity analysis in D45, D60, D90, and D120 organoids of COCO group (*n* = 27, 30, 17, 5, respectively) and COCO+IGF1 group (*n* = 27, 23, 10, 4, respectively) versus control retinal organoids (*n* = 17, 14, 11, 10, respectively). **d** Photoreceptor precursor cells between COCO group and control. **e** Immunohistochemistry with anti-RCVRN antibody on D45 and D60 organoids. Scale bar, 50 μm

The classic 3D differentiation system achieved retinalization through the combined effects of early Wnt pathway inhibition, the addition of extracellular matrix, and the activation of Hedgehog signaling [21]. COCO is a co-inhibitor of the Wnt, TGF-β, and BMP pathways that is to steer ESCs to differentiate into S cone cells, especially when combined with IGF1 [21–23]. To investigate whether COCO can replace the role of Wnt inhibitors in 3D differentiation, we first replaced IWR1e with COCO in culture medium for 12 days (Fig. 3a). We found that whether COCO was used alone or in combination with IGF1, the efficiency of organoid production was greatly reduced (Figure S2A). These organoids, which were similar to retinal organoids morphologically, only showed weak fluorescence in early-stage differentiation. After D45, organoid fluorescence faded away and eventually was absent. This result indicate that 3D differentiation treated with COCO (including combined with IGF1) cannot effectively produce retinal organoids.

Then, we investigated whether COCO, as an auxiliary additive, could increase the yield of precursors in 3D differentiation. We speculated that the timing of COCO in culture medium should be adapted to that of IWR1e, considering the relatively weak inhibition under the above comparison. To study whether long-term supplementation with COCO was more beneficial to 3D differentiation, we extended the supplementation time to 30 days. The overall fluorescence exhibited stronger intensity in the 12-day group than in the 30-day group in D30 organoids (Figure S2B). Moreover, the survival rate of organoids in the 30-day group was sharply reduced. There was no survival rate difference between the control and the 12-day group. This result indicates that long-term supplementation with COCO is not conducive to retinal organoid differentiation and photoreceptor precursor production.

Next, we compared the fluorescence intensity changes after COCO supplementation. Figure 3 b and d show that compared to the control group, the COCO group showed an increase in fluorescence intensity in the early stage of differentiation (35 and 45 days), while the combined treatment with COCO and IGF1 resulted in no fluorescence increase compared with the control. We performed further flow cytometry analysis on the COCO and control groups. An increased number of fluorescent cells appeared in organoids at D45 but not at D60 and D90. These results showed a consensus between fluorescence intensity and the number of fluorescent cells; that is, COCO can assist IWR1e in increasing photoreceptor precursor production in the early stage of 3D differentiation.

### Expression of signature genes in retinal organoids supplemented with COCO

In the presence of COCO, Wnt pathway component β-CATENIN (one of transducers) will be degraded in the cytoplasm, showing a typical Wnt pathway inhibitory. We first tested the expression of β-CATENIN (*CTNNB1*). Quantitative PCR results showed that β-CATENIN was significantly reduced its expression on D12 compared to the control group, demonstrating that COCO plays a role in 3D differentiation by inhibiting the Wnt pathway. There was no significant difference in β-CATENIN expression after D12, indicating the short-term inhibition of COCO.

**Fig. 4:**
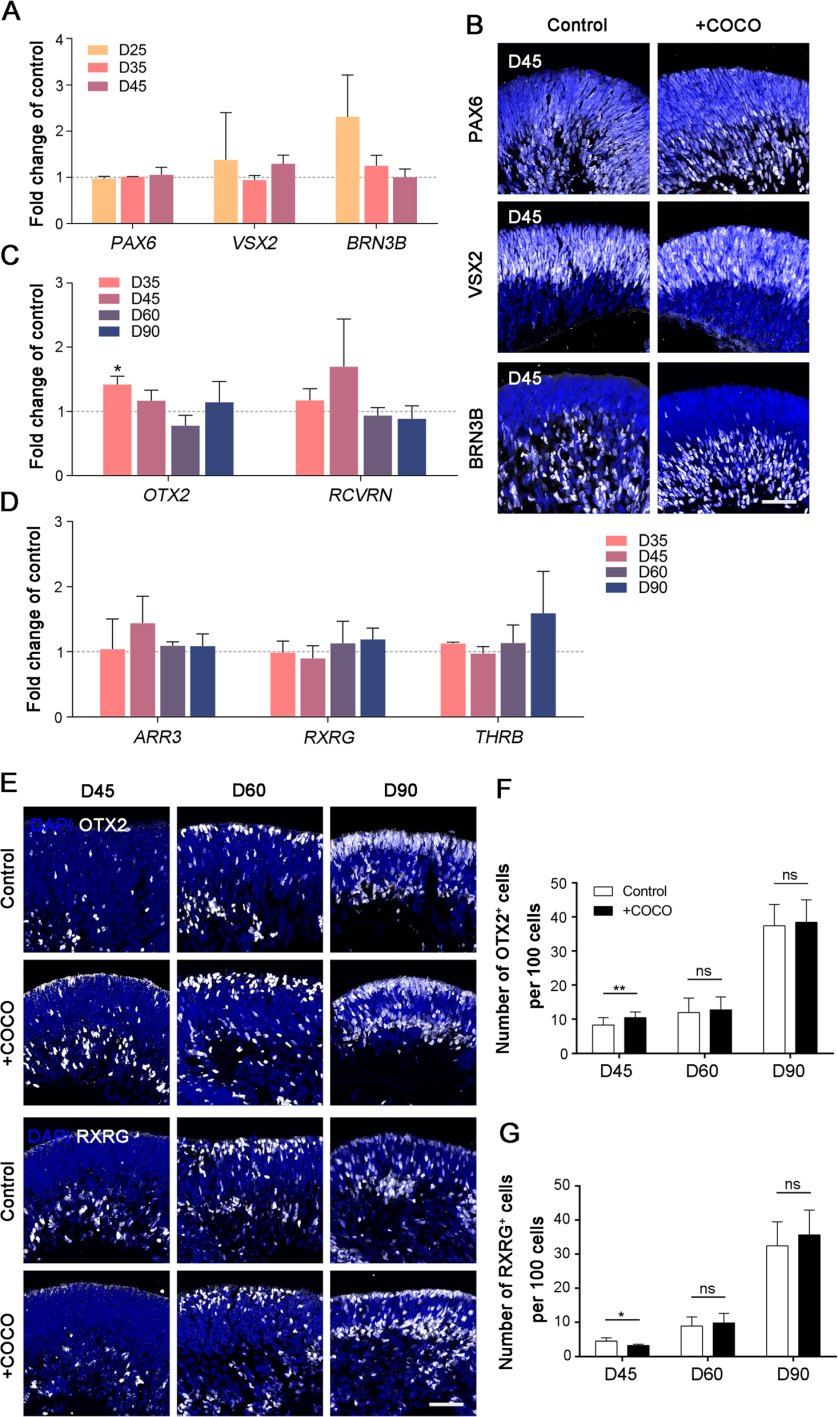
Characterization of marker gene expression in 3D organoids after COCO supplement. **a** Percentage mRNA expression of *PAX6*, *VSX2*, and *BRN3B* transcripts in early-stage differentiation analyzed by qPCR in COCO supplement and control. **b** Immunostaining of gene products showed in **a**. Scale bar, 50 μm. **c** Percentage mRNA expression of photoreceptor related *OTX2* and *RCVRN* transcripts. **d** Percentage mRNA expression of all subtypes of cones expressed *ARR3* and M-cone expressed *RXRG* and *THRB* transcripts in COCO supplement and control. Data are expressed as mean ± SEM. **e** Immunocytochemical analysis with anti-OTX2 and anti-RXRG. Scale bar, 50 μm. **f**, **g** Statistic analysis of OTX2^+^ and OTX2^+^ cells in organoids. *P < 0.05, **P < 0.01, ns, not significant

We examined gene expression during retinal organoid differentiation after COCO supplementation. In D25, D35, and D45 organoids, expression of retinal progenitor markers *PAX6* and *VSX2*, and retinal ganglion cell marker *BRN3B* did not significantly change compared with their expression in the control group (Fig. 4 a). Immunofluorescence staining of the three gene expression products showed neither cell distribution nor cell number changes, suggesting undifferentiated progenitor cells and retinal ganglion cells that were similar to those of the control group (Fig. 4b). *OTX2*, an upstream gene participated in photoreceptor development, only transiently increased its expression in D35 organoids, suggesting that COCO might be involved in *OTX2* regulation (Fig. 4c). In D35, D45, D60, and D90 organoids, neither the panphotoreceptor marker *RCVRN* nor the cone marker *ARR3* showed significant changes compared with the control (Fig. 4d). The M cone-related genes* RXRG* and *THRB* also maintained their expression levels after COCO supplementation. It is puzzling that the increased number of precursor cells in the early stage did not lead to a corresponding increase in gene expression related to photoreceptors. We assumed that COCO promoted a proportion of cells to enter into a photoreceptor precursor cell fate, while photoreceptor-related genes in each cell might be downregulated ultimately, resulting an unchanged mRNA level in bulk population of cells.

We further examined the spatial distribution of protein expression in organoids. At D45 of differentiation, the proportion of OTX2-positive cells increased while RXRG-positive cells decreased significantly, suggesting portion of cells changed their fates during early differentiation. There were no significant differences in D60 and D90 organoids on both kinds of cells. Combining the results of gene expression and immunostaining, we believe that COCO drives a part of cells into photoreceptor precursor fate at the early stage of differentiation.

### Increased number of cone photoreceptors in retinal organoids

**Fig. 5:**
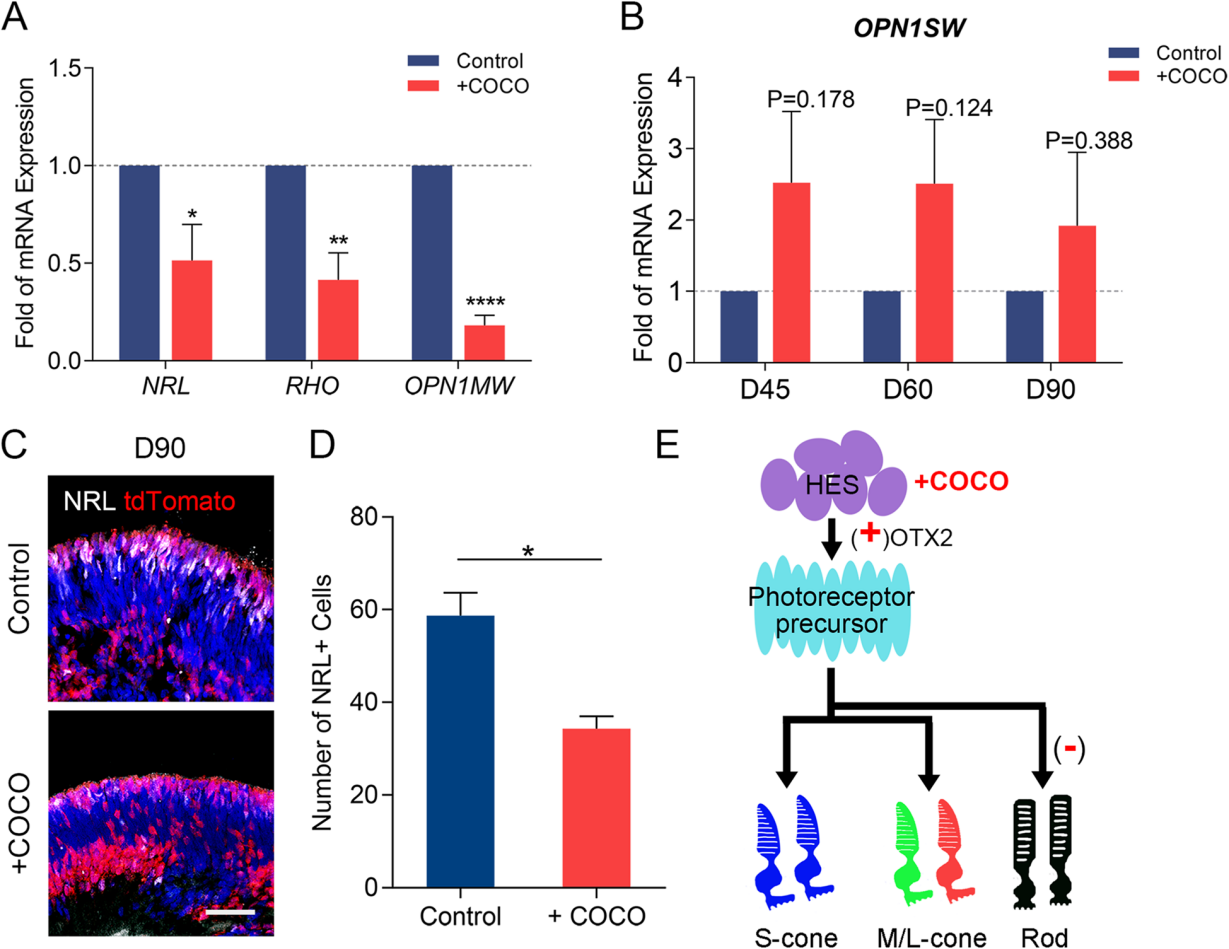
Effects on photoreceptors at middle developmental stage after COCO supplement. **a** Percentage mRNA expression of *NRL*, *RHO*, and *OPN1MW* transcripts in D90 organoids analyzed by qPCR in COCO supplement and control. **b** Percentage mRNA expression of *OPN1SW* transcripts in D45, D60, and D90 organoids. **c**, **d** Immunocytochemical analysis and quantification for NRL. Data are expressed as mean ± SEM. Scale bar, 50 μm. **e** The proposed effect of COCO at early developmental stage intervention in 3D retinalization. *P < 0.05

Research has shown that early supplementation with COCO inhibits multiple pathways (BMP, TGF-β, and Wnt) and guides the development of embryonic stem cells to differentiate in the direction of S cone cells [21]. To verify whether COCO has S cone guiding ability in this 3D differentiation and to assess photoreceptor precursor fate in later differentiation after COCO supplementation, we examined the expression of photoreceptor genes during maturation in retinal organoids. *NRL* and *RHO*, which are expressed in rod cells, were downregulated in D90 organoids (Fig. 5a). The number of NRL-positive cells also decreased in anti-NRL immunostaining experiments (Fig. 5c, d). Therefore, we concluded that the number of rod cells in the longer period is suppressed after COCO supplementation. The total number of photoreceptors and their precursor cells in D90 organoids did not differ after COCO supplementation (Fig. 3d). Therefore, the decrease in the number of rod cells means an increase in the number of cone cells at middle differentiation stage. There was no significant increase in the expression of the S cone opsin gene *OPN1SW* in D45, D60, and D90 organoids (Fig. 5b). At the same time, the expression of the M cone opsin gene *OPN1MW* decreased in D90 organoids. Considering the existence of the S cone default pathway in photoreceptor development [24, 25], we speculated that the downregulated *OPN1MW* may suggest a muted process of S cone development into M cone cells after COCO supplementation, or it may suggest slowed maturation of M cone cells (Fig. 5e).

## Discussion

In vitro organoid differentiation technology can recapitulate the process of organ development and has attracted widespread attention for its ability to simulate disease progression and aid in drug screening, especially in the study of human development and human diseases [26, 27]. Fluorescently labeled cell lines are one of the most powerful tools in this process. Such lines not only can help verify the effectiveness of the differentiation method and obtain an optimized differentiation scheme but also can enable many tools for subsequent research, including cell sorting, gene expression analysis, and cell transplantation. In this study, to improve the differentiation efficiency of photoreceptor precursor cells, the reporter gene *tdTomato* was integrated into the marker gene *CRX*, which is expressed in photoreceptor precursor cells, resulting in an engineered ESC line. During 3D retinal organoid differentiation, the fluorescence intensity was monitored to indicate the efficiency of precursor cell production. As a result, fluorescence successfully tracks precursor cells during subsequent differentiation without retinalization disturbance. By supplying COCO to inhibit the Wnt, TGF-β, and BMP signaling pathways, we observed an increase in precursor cells in the early stage of retinal organoids. In the long run, the number of rod cells declines after COCO intervention in 3D differentiation.

With a deep understanding of retinal development, remarkable progress has been made in differentiating stem cells into a retinal lineage using in vitro culturing systems [10, 28–30]. In earlier studies of 2D adhesion culturing, it was found that in serum-free systems, early signal pathway interventions can gradually successfully guide ESCs to differentiate into retinal cells [31]. After coculture with real retinal cells or further intervention with additives such as retinoic acid, these differentiated cells can begin to express mature photoreceptor genes [32]. However, for retinal cells differentiated in vitro, there was difficulty in getting the cells to exhibit the typical morphology and physiological functions of real retinal cells until the emergence of 3D differentiation systems. In fact, in vitro ESCs immediately exhibit self-adhesive and self-organized differentiation characteristics, which reflect natural embryonic development. Recent studies have proven that physiological differences play a role in cell therapy, which makes transplanted cells difficult to integrate into the host retina in a robust and efficient manner [4, 33]. Although the potential rule of self-organization in 3D differentiation has not been fully understood, developmental biology has shown that a large number of signaling pathways (FGF, IGF, BMP, Nodal, Wnt, and Notch) drive this process for precise regulation [24, 34–37].

Although lacking the Wnt antagonist Dkk1 and the BMP antagonist noggin in 2D differentiation, the addition of COCO can significantly increase the expression of photoreceptor-related genes, especially when COCO is used in combination with IGF1 [21]. However, in the 3D differentiation system in this study, no stable fluorescence expression of retinal organoids could be obtained either by supplementation with COCO alone or in combination with IGF1. At the same time, the survival rate of retinal organoids significantly decreased, indicating that the COCO effect is probably not as strong as that of the Wnt inhibitor IWR1e in the classic 3D culturing system. Our results demonstrate that COCO can only act as an auxiliary additive to IWR1e to contribute to further pathway inhibition.

The process of in vitro differentiation mimics real retinal development. During the transformation of PSCs into the retina, early inhibition of the Wnt pathway can promote ectoderm development, with obvious PAX6-positive cells appearing [20]. However, the loss of β-catenin, a Wnt signaling effector, disrupts the radial arrangement of retinal progenitor cells, leading to the severe retina destruction as well [38]. Therefore, it is obviously inappropriate to blindly inhibit the Wnt signaling pathway at different stages in 3D differentiation. The same rules were also appropriate for the BMP pathway. Considering the widespread presence of retinal progenitor cells and ganglion cells in D30 organoids, activation of the BMP pathway and Wnt pathway in this stage is necessary. Therefore, in this study, COCO was only used to temporarily and simultaneously inhibit the Wnt pathway in the original 3D culture system. It turns out that addition of COCO for a long duration in culturing is detrimental to organoid survival.

Even as a supplemental additive, the COCO effects were relatively weak considering the upregulation of *OTX2* only in the early stage and the downregulation of rod-related genes and the M-cone opsin gene *OPN1MW* in the middle stage. The strategy of reporter gene knock-in carried out in this study may be another reason for this weak result. *tdTomato* is inserted after the promoter of *CRX* exon 2 and ends with the poly-A tail. This insertion strategy allows the engineered allele to express *tdTomato* but not *CRX* via the *CRX* promoter. Like most knock-in strategies in organoid research, this post-promoter insertion strategy provided a relatively robust tracking tool but at the cost of losing the expression of one allele. Therefore, we created a *CRX* monoallelic knockout cell line, which resulted in a decrease in *CRX* expression during differentiation. In the *CRX* monoallelic knockout mice, photoreceptor outer segment lengths were shortened and S cone opsin was slightly decreased at P14, while no embryonic data were available for reference [39]. A *CRX* mutation, which is the equivalent of a monoallelic knockout in humans, also constitutes a clear cause of blindness [40]. We speculate that haploinsufficiency of CRX may affect photoreceptor development in the early stage of disease. For these reasons, we do not rule out the weak effect of COCO in 3D differentiation when considering insufficient doses of CRX due to *tdTomato* knock-in.

## Conclusion

In summary, this study produced a fluorescently labeled stem cell line enabling tracking of cells expressing *CRX* and demonstrated that this system can be used to optimize differentiation scheme for monitoring photoreceptor precursor cell production under 3D retinal organoid differentiation. Through screening, we proved that the addition of COCO protein can increase the number of precursor cells in the early stage of differentiation. Obviously, the fluorescence knock-in strategy can be expected to be further broadened to play a greater role in the study of multicellular biology.

## Supplementary information

**Additional file 1 :** Figure S1. Characterization of CRXp-tdTomato. a Polymerase chain reaction of CRXp-tdTomato clones with primers spanning the integration site to assess whether the cassette had integrated at the correct site. b Karyotype analysis of CRXp-tdTomato. c Immunohistochemical analysis of alkaline phosphatase. Scale bar, 400 μm. d Immunocytochemistry of marker proteins for α-fetoprotein [AFP] (marker of endoderm), α-smooth muscle actin [α-SMA] (marker of mesoderm) and GFAP (marker of ectoderm). Scale bar, 100 μm. Figure S2. 3D retinal organoids formed by COCO supplement schemes. a Morphology of retinal organoids after COCO with or without IWR1e and IGF1. Scale bar, 2000 μm. b Comparison of COCO inducing efficiency between 12-day-supplement and 30-day-supplement versus control group on D30 organoids. c Quantification of survival rate of organoids in panel B. e Fold change of *CTNNB1* expression. *P < 0.05.

## Data Availability

The datasets used and/or analyzed during the current study are available from the corresponding author on reasonable request.

## Abbreviations

ESCs: Embryonic stem cells
PSCs: Pluripotent stem cells
TGF-β: Transforming growth factor β
BMP: Bone morphogenetic protein
IGF1: Insulin-like growth factor 1
GSK3: Glycogen Synthase Kinase 3
FGFR: Fibroblast growth factor receptor
CRX: Cone-Rod homeobox
NRL: Neural retina leucine zipper
