# Supplementary material for: COCO enhances the efficiency of photoreceptor precursor differentiation in early human embryonic stem cell-derived retinal organoids
Source: Stem Cell Res Ther. 2020 Aug 24;11:366. doi: 10.1186/s13287-020-01883-5 (PMC7444242; doi:10.1186/s13287-020-01883-5)
Supplement: Supplementary file 1 — Additional file 1 : Figure S1. Characterization of CRXp-tdTomato. a Polymerase chain reaction of CRXp-tdTomato clones with primers spanning the integration site to assess whether the cassette had integrated at the correct site. b Karyotype analysis of CRXp-tdTomato. c Immunohistochemical analysis of alkaline phosphatase. Scale bar, 400 μm. d Immunocytochemistry of marker proteins for α-fetoprotein [AFP] (marker of endoderm), α-smooth muscle actin [α-SMA] (marker of mesoderm) and GFAP (marker of ectoderm). Scale bar, 100 μm. Figure S2. 3D retinal organoids formed by COCO supplement schemes. a Morphology of retinal organoids after COCO with or without IWR1e and IGF1. Scale bar, 2000 μm. b Comparison of COCO inducing efficiency between 12-day-supplement and 30-day-supplement versus control group on D30 organoids. c Quantification of survival rate of organoids in panel B. e Fold change of CTNNB1 expression. *P < 0.05. [file 13287_2020_1883_MOESM1_ESM.docx]

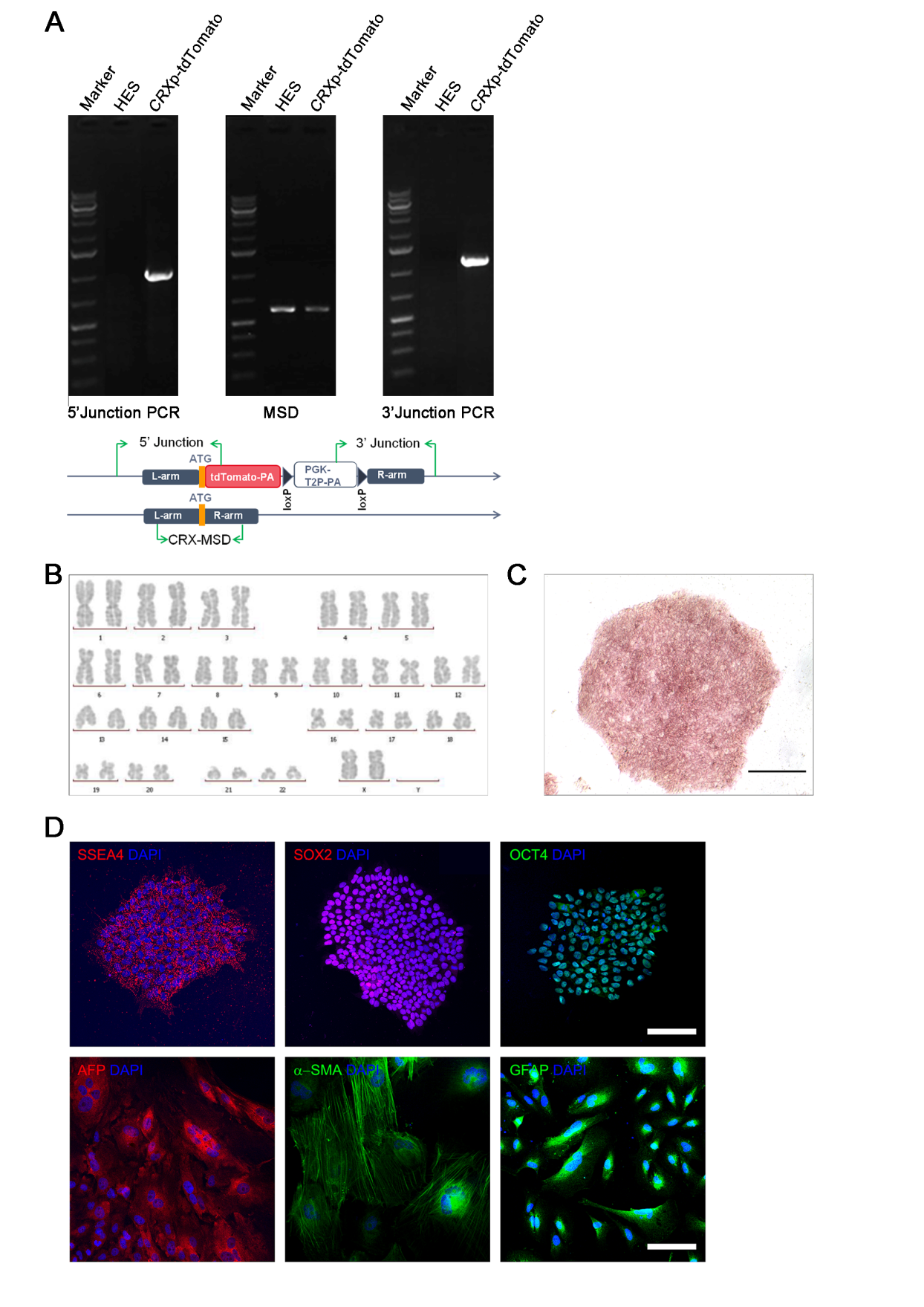


**FIGURE S1. Characterization of CRXp-tdTomato.** **a** Polymerase chain reaction of CRXp-tdTomato clones with primers spanning the integration site to assess whether the cassette had integrated at the correct site. **b** Karyotype analysis of CRXp-tdTomato. **c** Immunohistochemical analysis of alkaline phosphatase. Scale bar, 400 μm. **d** Immunocytochemistry of marker proteins for α-fetoprotein [AFP] (marker of endoderm), α-smooth muscle actin [α-SMA] (marker of mesoderm) and GFAP (marker of ectoderm). Scale bar, 100 μm.


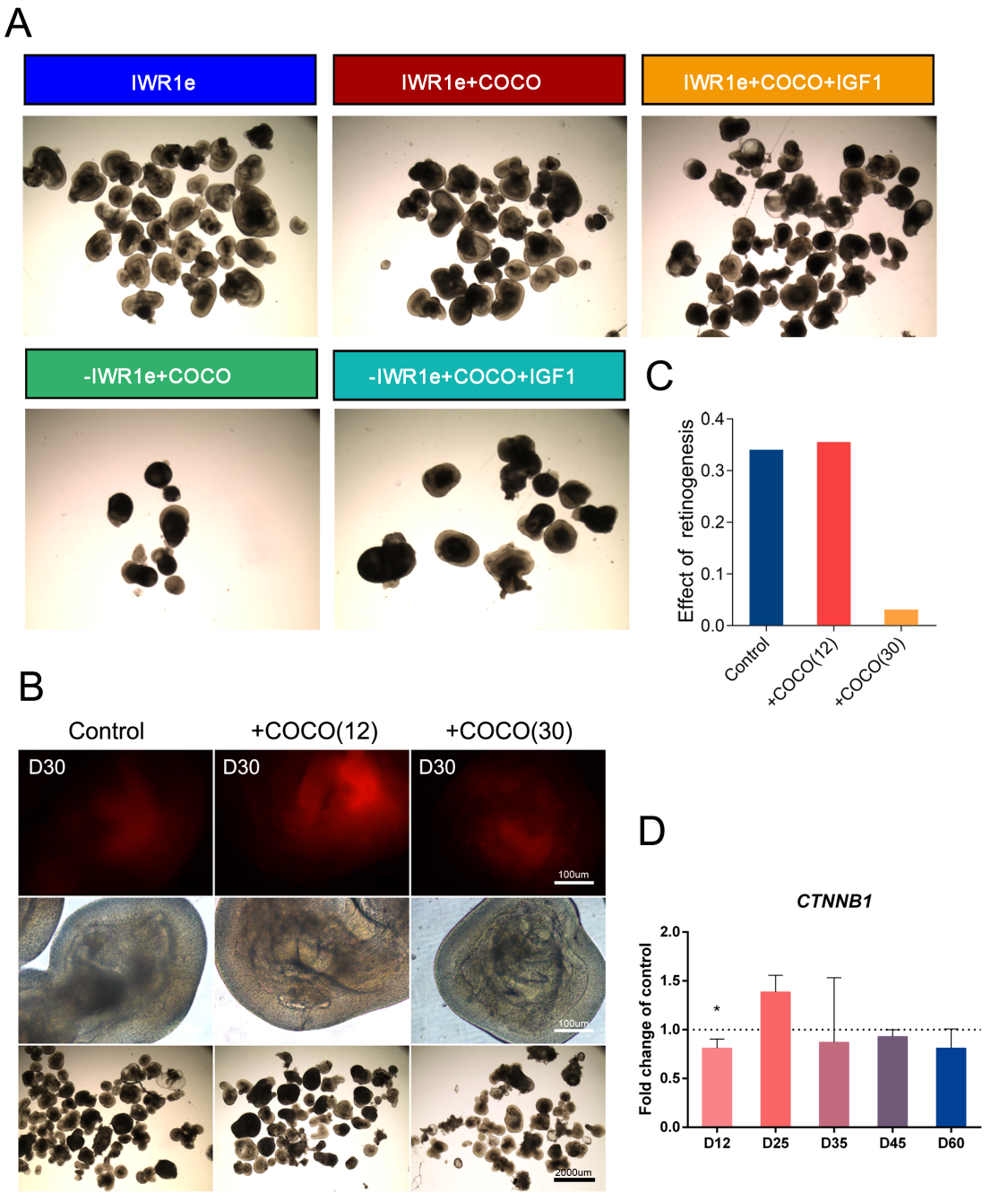


**FIGURE S2. 3D retinal organoids formed by COCO supplement schemes. a** Morphology of retinal organoids after COCO with or without IWR1e and IGF1. Scale bar, 2000 μm. **b** Comparison of COCO inducing efficiency between 12-day-supplement and 30-day-supplement versus control group on D30 organoids. **c** Quantification of survival rate of organoids in panel B. **e** Fold change of *CTNNB1* expression. **P* < 0.05.
